# Dexmedetomidine Attenuates Apoptosis and Neurological Deficits by Modulating Neuronal NADPH Oxidase 2-Derived Oxidative Stress in Neonates Following Hypoxic Brain Injury

**DOI:** 10.3390/antiox11112199

**Published:** 2022-11-07

**Authors:** Xiaohui Chen, Dongtai Chen, Pinzhong Chen, Andi Chen, Jianhui Deng, Jianjie Wei, Weian Zeng, Xiaochun Zheng

**Affiliations:** 1 Department of Anesthesiology, Shengli Clinical Medical College, Fujian Medical University, Fujian Provincial Hospital, Fuzhou 350001, China; 2 Department of Anesthesiology, Sun Yat-sen University Cancer Center, State Key Laboratory of Oncology in South China, Guangzhou 510060, China; 3 Fujian Provincial Key Laboratory of Emergency Medicine, Fujian Provincial Key Laboratory of Critical Care Medicine, Fujian Provincial Co-Constructed Laboratory of “Belt and Road”, Fuzhou 350001, China

**Keywords:** dexmedetomidine, NADPH oxidase 2, oxidative stress, neuronal apoptosis, neurological deficits, hypoxic brain injury, neonates

## Abstract

Hypoxic–ischemic brain injury is an important cause of neonatal neurological deficits. Our previous study demonstrated that dexmedetomidine (Dex) provided neuroprotection against neonatal hypoxic brain injury; however, the underlying mechanisms remain incompletely elucidated. Overactivation of NADPH oxidase 2 (NOX2) can cause neuronal apoptosis and neurological deficits. Hence, we aimed to investigate the role of neuronal NOX2 in Dex-mediated neuroprotection and to explore its potential mechanisms. Hypoxic injury was modeled in neonatal rodents in vivo and in cultured hippocampal neurons in vitro. Our results showed that pre- or post-treatment with Dex improved the neurological deficits and alleviated the hippocampal neuronal damage and apoptosis caused by neonatal hypoxia. In addition, Dex treatment significantly suppressed hypoxia-induced neuronal NOX2 activation; it also reduced oxidative stress, as evidenced by decreases in intracellular reactive oxygen species (ROS) production, malondialdehyde, and 8-hydroxy-2-deoxyguanosine, as well as increases in the antioxidant enzymatic activity of superoxide dismutase and glutathione peroxidase in neonatal rat hippocampi and in hippocampal neurons. Lastly, the posthypoxicneuroprotective action of Dex was almost completely abolished in NOX2-deficient neonatal mice and NOX2-knockdown neurons. In conclusion, our data demonstrated that neuronal NOX2-mediated oxidative stress is involved in the neuroprotection that Dex provides against apoptosis and neurological deficits in neonates following hypoxia.

## 1. Introduction

Neonatal hypoxic–ischemic brain injury, a common clinical problem caused by perinatal hypoxia, is associated with high mortality and lifelong neurological and cognitive deficits in infants and children [1,2]. The pathophysiology of hypoxic brain injury is highly complex and not completely understood. Studies have indicated that oxidative stress, inflammation, and excitotoxicity are implicated in the pathological process of brain injury [3,4]. Among these factors, oxidative stress has been regarded as a significant contributor to the initiation and progression of hypoxic brain injury in neonates [5,6]. Oxidative stress represents an imbalance between pro-oxidants and antioxidants that is usually caused by excessive reactive oxygen species (ROS). It is well documented that increased levels of ROS can cause oxidative damage to cell membranes, nucleic acids, and proteins, leading to neuronal apoptosis and death in various hypoxia-related neurological disorders [7,8,9]. Thus, effective prevention and treatment of excessive ROS generation has become the focus of research on neonatal hypoxic brain injury.

Although the mitochondria are thought to be the main source of ROS production, numerous studies have shown that NADPH oxidase 2 (NOX2) is abundantly expressed in cortical and hippocampal neurons, and this molecule is regarded as another important source of ROS under physiological and pathological conditions [10]. Experimental studies have demonstrated that excessive NOX2 activation contributes significantly to oxidative damage to neurons and other cell types in traumatic, ischemic, and degenerative injuries of the central nervous system (CNS) [11,12,13]. Specifically, pharmacological inhibition or genetic deletion of NOX2 using currently available methods was shown to protect against oxidative stress, progressive neurodegeneration, and long-term neurocognitive deficits in multiple neurological disorders [14]. Our previous studies also demonstrated that neuronal NOX2-derived ROS resulted in mitochondrial dysfunction and apoptosis in in vitro models of hypoxia and oxidative stress [15,16]. Hence, neuronal NOX2 may be a promising therapeutic target for improving neurological deficits in neonates following hypoxia.

Dexmedetomidine (Dex), a highly selective *α*-2 adrenergic receptor agonist that provides sedative, analgesic, and anxiolytic effects without obvious respiratory depression, is being increasingly used in pediatric anesthesia and intensive care [17,18]. Dex has been reported to provide neuroprotection against neuronal apoptosis and neurological deficits in animal models of hypoxic brain injury and anesthetic neurotoxicity in newborns [19,20,21]. A recent study [22] from our laboratory demonstrated that Dex ameliorates hypoxia-induced synaptic and cognitive deficits in neonatal rats, partly by inhibiting microglial activation and neuroinflammation. Furthermore, several studies have found that these antioxidant, anti-inflammatory, and antiapoptotic effects of Dex are closely related to the activation of the *α*-2-adrenergic receptor and binding to the imidazoline receptor [23,24]. However, the underlying mechanisms responsible for the neuroprotection of Dex remain incompletely elucidated. Therefore, a detailed understanding of the molecular mechanism underlying Dex–induced-neuroprotection would be beneficial for the future development of novel therapeutic strategies against neonatal hypoxic brain injury.

Accordingly, this study aimed to further investigate the mechanism of Dex on neurological deficits caused by neonatal hypoxia, and the effects of Dex on neuronal NOX2 activation and oxidative stress were evaluated. In addition, the effective pharmacological targeting of Dex was further determined by using mice or cultured hippocampal neurons lacking NOX2.

## 2. Materials and Methods

### 2.1. Animals

Pregnant Sprague–Dawley (SD) rats and their offspring were purchased from the Experimental Animal Center of Sun Yat-sen University and Fujian Medical University. C57BL/6J wild-type (WT) and NOX2-deficient mice (NOX2^−/−^, B6.129S-Cybbtm1Din/J, stock number: 002365, Jackson Laboratories, Bar Harbor, ME, USA) were used in this study. All animals were kept under a 12:12 light–dark cycle in an animal room maintained at 24–25 °C, and food and water were provided ad libitum. We conducted our animal experiment in compliance with the Laboratory Animal Care and Use Guide (National Research Council Committee, 2011).

### 2.2. Neonatal Hypoxia and Dex Treatments In Vivo

For the in vivo studies, rodent pups at postnatal day 3 (PND 3) were placed into a hypoxic chamber (Model: MCO 18 M; Sanyo Biomedical Electrical Co., Ltd., Tokyo, Japan) and exposed to a continuous flow of a humidified 5% O_2_ and 95% N_2_ gas mixture at 37 °C for 2 h as previously described [22]. Pups were then given 30 min to recover before being returned to their mother. Meanwhile, age- and sex-matched littermates were used as normoxic controls.

**Figure 1:**
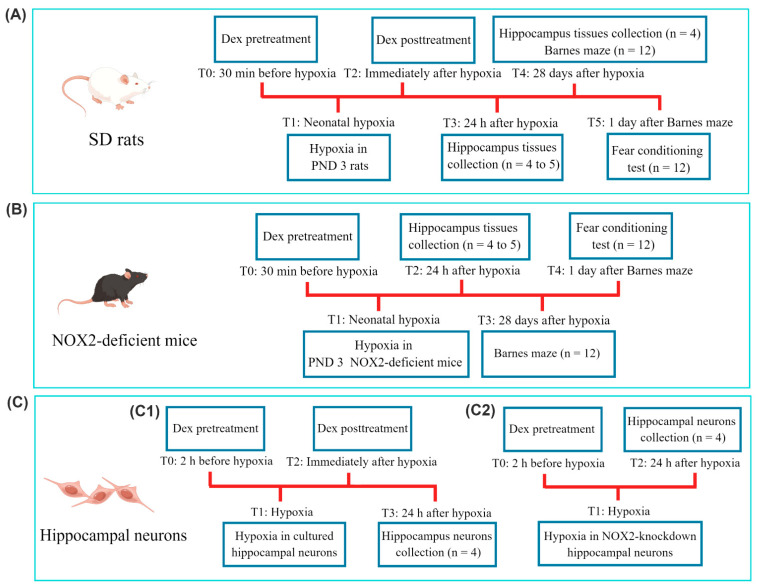
Schematic diagram summarizing the timeline of the experimental protocol. Procedures at different time points (T0 to T5) are described. (**A**) SD rats were subjected to hypoxia and Dex treatment on PND 3. (**B**) NOX2-deficient mice were subjected to hypoxia and Dex treatment on PND 3. (**C**) For the in vitro experiments, (**C1**) cultured hippocampal neurons were subjected to hypoxia and Dex treatment. (**C2**) Cultured hippocampal neurons were transfected with NOX2-siRNA or scrambled siRNA, and then subjected to hypoxia and Dex treatment. The n number in the diagram represents the number of animals per group or the number of experimental repeats. PND, postnatal day; SD rats, Sprague–Dawley rats; Dex, dexmedetomidine.

The pups were randomly assigned into four groups: (1) control rats receiving saline (Control group), (2) hypoxic rats receiving saline (Hypoxia group), (3) hypoxic rats pretreated with Dex (Pre-Dex + Hypoxia group), and (4) hypoxic rats pretreated with Dex (Post-Dex + Hypoxia group). Next, Dex (25 μg/kg, Sigma-Aldrich, St. Louis, MO, USA) was applied intraperitoneally 30 min before the onset of hypoxia or immediately after hypoxia. The dosage and timing of the administration of Dex were determined on the basis of previous neuroprotection research, including ours [22,25,26]. Both the control and hypoxia groups received equal volumes of saline. The levels of neuronal NOX2 expression and NOX activity, oxidative stress, and neuronal survival and apoptosis in the immature hippocampus were evaluated 1 d after hypoxia. Neurological function and neuronal survival were evaluated 28 d after hypoxia (Figure 1A).

Furthermore, to clarify the effective pharmacological target of Dex in neonatal hypoxic brain injury, we validated our findings by treating wild-type and NOX2-deficient mice with hypoxia and Dex. Neurological function and oxidative stress levels were evaluated following neonatal hypoxia (Figure 1B).

### 2.3. In Vitro Hypoxia and Dex Treatment

In vitro hypoxia studies were performed as previously described [27]. Primary hippocampal neurons were maintained in a closed hypoxia chamber (model: MCO 18 M) flushed with 1% O_2_, 5% CO_2_, and 94% N_2_ for 12 h at 37 °C. Next, the neurons were cultured at 37 °C for 24 h in a 5% CO_2_ and 95% air environment. Neurons cultured under normoxic conditions were used as a negative control.

The neurons were also divided into four groups: (1) normoxic cells (Control group), (2) hypoxic cells (Hypoxia group), (3) hypoxic cells pretreated with Dex (Pre-Dex + Hypoxia group), and (4) hypoxic cells post-treated with Dex (Post-Dex + Hypoxia group). Dex (1 μM) was added to the culture media 2 h before hypoxia or immediately after hypoxia. The concentration and timing of the Dex application were determined as described in prior studies [15,22]. The levels of neuronal NOX2 expression and NOX activity, oxidative stress, mitochondrial function, and apoptosis were detected 24 h after hypoxia (Figure 1C).

In order to further investigate the role of neuronal NOX2 in Dex-mediated neuroprotection, we knocked down NOX2 expression using a small interfering RNA (siRNA). Next, the levels of intracellular ROS production and apoptosis were evaluated (Figure 1C).

### 2.4. Primary Hippocampal Neuronal Cultures

As previously described [22], P0–P1 SD rat pups were used for the preparation of primary hippocampal neuronal cultures. Briefly, we dissected the hippocampi and digested them with 2 mg/mL papain at 37 °C for 30 min. Cells were plated at a density of 1 × 10^6^ cells/well onto poly-D-lysine-coated 6-well plates in neurobasal medium (GIBCO, Billings, MT, USA) supplemented with 0.5 mM glutamine (HyClone, Logan, UT, USA) and 2% B27 (GIBCO). Three days after the medium was replaced, half was replaced with fresh medium, and it took seven to eight days for the cells to differentiate before they were used. The purity of the neurons was >95%, as quantified by immunofluorescence using anti-NeuN antibodies.

### 2.5. RNA Interference

For in vitro RNA interference (RNAi), we purchased siRNA from GenePharma (Shanghai, China) to specifically knock down the expression of the NOX2 gene in primary hippocampal neurons. Lipofectamine RNAiMAX (Invitrogen, Carlsbad, CA, USA) was used to transfect the cells with siRNA targeting NOX2 or scrambled siRNA as a control. After transfection for 48 h, NOX2 mRNA expression levels were determined using qRT-PCR.

### 2.6. Behavioral Tests

The Barnes maze and fear conditioning test were performed in order to evaluate the cognitive functions of animals following acute hypoxia and the Dex treatment.

In animals, the Barnes maze is widely used to study hippocampus-dependent spatial memory and learning [28,29]. The maze was composed of a circular platform (122 cm in diameter) and 18 circular holes (10 cm in diameter). A loud sound (85 dB) accompanied by a bright light (200 W) on a platform was used to encourage the animals to find the target area. The animals were allowed 3 min to find the target hole on 4 consecutive days of training trials on PND31–PND34. On day 1 (PND35) and day 8 (PND42) after the training trials, the escape box was removed and the animals were given 90 s to find it (probe trials). Video recordings were taken during each session using an overhead camera from ANY-maze (Stoelting Co., San Diego, CA, USA).

The animals were then tested for fear conditioning 1 d after the Barnes maze as previously described [29]. Briefly, each animal was placed in a conditioning chamber; after a 3 min exploratory period, the animals were exposed to 3 tones (4.5 kHz, 60 dB, 30 s), each of which was paired with a foot shock (1 mA, 5 s). Twenty-four hours later, the animal was returned to the same chamber for 8 min without tones or shocks to assess their contextual fear conditioning. We divided the time into 8-s intervals and recorded how many of these intervals included freezing behavior. After two hours, each animal was placed in a new chamber that differed from the first chamber in smell and other contextual cues. To measure cued fear conditioning, freezing behavior was recorded during a 3-min period without any further presentation of stimuli. Video recordings of freezing behavior were made, and an observer blinded to the animal groups scored them later.

### 2.7. Preparation of Hippocampal Brain Slices

Hippocampal slices were prepared as previously described. All animals were anesthetized before being perfused with a phosphate buffer and phosphate-buffered 4% paraformaldehyde. We removed the brains from the animals and immersed them in 4% paraformaldehyde overnight. Then, 5-μm-thick brain sections were cut from the tissue after dehydration and paraffin embedding. Hippocampal slices were used for Nissl, TUNEL, and immunofluorescence staining according to standard procedures in some experiments.

### 2.8. Histopathological Examination in the Hippocampus

Nissl staining was performed in order to assess neuronal survival and morphological changes in the hippocampus at 1 d or 28 d after neonatal hypoxia, as previously described [30]. First, we dipped and stained these brain sections in 1% toluidine for 10 min. Next, they were rinsed with distilled water, dehydrated with ethanol (70%, 80%, 95%), cleared with xylene, and mounted with neutral resin. Two individuals, who were blinded to the experimental treatments, inspected the hippocampal CA1 regions at 400× magnification to count Nissl-positive neurons.

A TUNEL assay was used to evaluate the hippocampal neuronal apoptosis 1 d after neonatal hypoxia, as previously described [30]. TUNEL staining revealed that the apoptotic islet cells had dark brown nuclei, indicating that they were apoptotic. Hippocampal CA1 regions were counted at 400× magnification by two blinded individuals in order to determine the number of TUNEL-positive neurons. The apoptosis index (%) was calculated by dividing the number of TUNEL-positive cells by the total number of cells.

### 2.9. Assessment of Oxidative Stress

The quantities of malondialdehyde (MDA, a marker of lipid peroxidation) and 8-hydroxydeoxyguanosine (8-OHdG, a marker of oxidative DNA damage) and the activity levels of superoxide dismutase (SOD) and glutathione peroxidase (GSH-Px) in the hippocampus were assessed with assay kits (Nanjing Jiancheng Bioengineering Institute, Nanjing, China). In brief, MDA content was assessed using the thiobarbituric acid (TBA) method, SOD activity was determined using a WST-1 assay, and GSH-Px activity was determined using a UV colorimetric assay.

Intracellular ROS production was detected in cultured hippocampal neurons using the DCFH-DA probe (Beyotime, Shanghai, China). We plated 1 × 10^4^ cells per well in 24-well plates, and the cells were incubated in the dark for 30 min with DCFH-DA (10 μM). Inverted fluorescence microscopy (DMI4000B, Leica, Wetzlar, Germany) was used to capture the images, and the intensity of the fluorescence was analyzed using ImageJ (BX50-FLA, Olympus, Tokyo, Japan).

### 2.10. Immunofluorescence Staining of NOX2

Coronal hippocampal sections and cultured hippocampal neurons were prepared for immunofluorescence staining as previously described. In brief, sections were deparaffinized and dehydrated, and the antigens were extracted. Next, the sections were incubated with anti-NOX2 (1:50, Santa Cruz Biotech, Santa Cruz, CA, USA) and anti-NeuN antibodies (1:200, Cell Signaling Technology, Inc., Danvers, MA, USA) overnight, followed by a secondary antibody. Fluorescence microscopy (IX71, Olympus, Tokyo, Japan) was used to capture the images, and the intensity of fluorescence was analyzed using ImageJ.

Cultured hippocampal neurons were seeded at 2 × 10^4^ cells per well on 8-well chamber slides (Millipore, Carrigtwohill, Ireland), then 4% paraformaldehyde was used to fix the cells and 3% BSA in PBS was used to block them. Following incubation with the anti-NOX2 antibody (1:50, Santa Cruz), the cells were washed with PBS and then stained with Alexa 546-labeled secondary antibodies. A counterstain of DAPI (Beyotime) was applied to the nuclei of the cells. Images were captured using a confocal laser scanning microscope (FV10i, Olympus, Tokyo, Japan).

### 2.11. Measurement of NOX Activity

As described previously [22], cytochrome c reduction assays were used to measure NOX activity in vivo and in vitro. Briefly, hippocampal tissues or cultured hippocampal neuron lysates were incubated with oxidized cytochrome c at 37 °C for 3 min in a quartz cuvette. The samples were then incubated for 15 min with a NOX substrate added to the reaction mixture. A spectroscopic measurement at 550 nm was used to monitor the reduction in cytochrome c. NOX activity was expressed as nmol/min/mg protein.

### 2.12. Western Blotting Analysis

Western blot assays were performed as previously described [22]. Briefly, hippocampal tissues and hippocampal neurons were lysed using a RIPA lysis buffer to isolate proteins. Equal amounts of protein (40 μg) were loaded onto 12% SDS-polyacrylamide gels, followed by transfer to PVDF membranes. Subsequently, the membranes were incubated with primary antibodies against cleaved caspase-3 (1:500, Cell Signaling), Bax (1:1000, Cell Signaling), Bcl-2 (1:1000, Cell Signaling), NOX2 (1:200, Santa Cruz), 4-hydroxynonenal (4-HNE, 1:1000, Abcam, Cambridge, UK), and GAPDH (1:2000, Cell Signaling). The blots were developed with a chemiluminescence enhancement reagent and detected by an image analyzer (Bio-Rad Laboratories, Hercules, CA, USA). The results are expressed as a percentage of the control values.

### 2.13. Flow Cytometric Analysis of Apoptosis

Apoptosis was analyzed by flow cytometry using Annexin V-FITC/PI staining (Beyotime). Briefly, we collected, washed, and resuspended hippocampal neurons in 100 µL of a binding buffer containing 5 µL PI and 5 µL annexin V-FITC for 20 min. Flow cytometry (BD Bioscience, San Jose, CA, USA) was then used to measure neuronal apoptosis.

### 2.14. Assessment of Mitochondrial Membrane Potential

Mitochondrial membrane potential (MMP) was detected using JC-1 probes (Beyotime). The monomeric form of JC-1 emits green fluorescence at a low MMP, while the aggregate form emits red fluorescence at a higher MMP. After the corresponding treatments, a JC-1 dye solution was added to the cells for 20 min at 37°C, followed by the JC-1 dye buffer twice. Next, inverted fluorescence microscopy (Leica) was used to capture the images. The MMP was represented by the ratio of red to green fluorescence.

### 2.15. Statistical Analysis

Analysis of the data was carried out with SPSS 22.0 software (SPSS Inc., Chicago, IL, USA). We expressed the results as the mean ± standard deviation (SD). A Shapiro-Wilk normality test was performed to evaluate the normality of the data’s distribution. Multiple group comparisons were made by using one-way analysis of variance (ANOVA), followed by Tukey’s test if the data were normally distributed. When they were not normally distributed, a Kruskal-Wallis test was conducted. An analysis of the Barnes maze training data was performed using two-way repeated-measures ANOVA and Tukey’s test. Differences were considered statistically significant at a *p* value of <0.05.

## 3. Results

### 3.1. Dex inhibited NOX2 Activation and Oxidative Stress in Rat Hippocampus and Cultured Hippocampal Neurons Following Hypoxia

**Figure 2:**
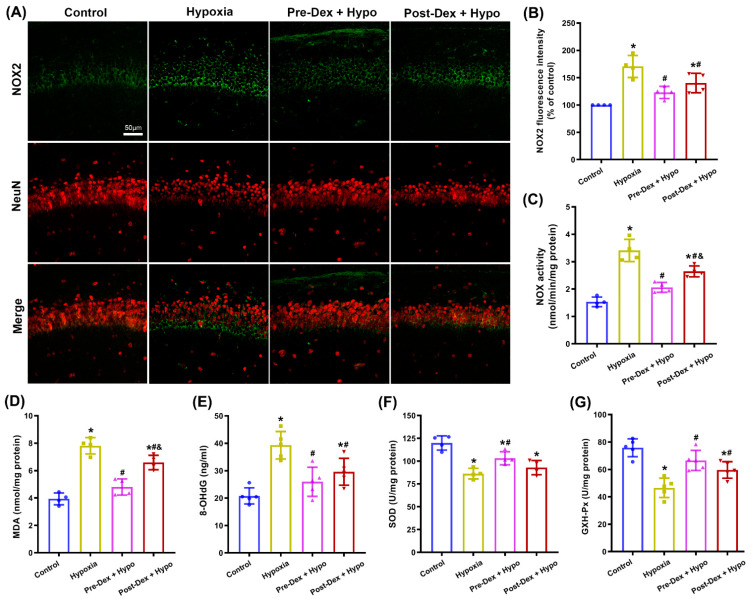
The effect of Dex on NOX2 activity and oxidative stress in the hippocampus following neonatal hypoxia. (**A**) Representative immunofluorescence images of NOX2 (green) and NeuN (red) in the hippocampal CA1 region. (**B**) The intensity of NOX2 fluorescence was quantified using ImageJ software. (**C**) A cytochrome c reduction assay was used to measure NOX activity. (**D**) MDA levels were assessed using the TBA colorimetric method. (**E**) DNA 8-OHdG levels were determined using an 8-OHdG ELISA kit. (**F**) SOD activity was assessed using the WST-1 method. (**G**) GSH-Px activity was assessed using the UV colorimetric assay. Results are expressed as mean ± SD (*n* = 4–5 in each group). Each animal data point in the bar graph is also presented. * *p* < 0.05 versus the Control group; ^#^ *p* < 0.05 versus the Hypoxia group; ^&^ *p* < 0.05 versus the Pre-Dex group. Hypo, hypoxia; Pre-Dex, pretreatment with dexmedetomidine; Post-Dex, post-treatment with dexmedetomidine.

In neurons, overactivation of NOX2 is thought to be involved in oxidative damage and neurodegeneration in various neurological diseases. In order to confirm whether the protective effects of Dex were correlated with the modulation of NOX2 activity, we first evaluated the extent of neuronal NOX2 activation by assessing the expression of NOX2 protein and NOX activity in the neonatal rat hippocampus and cultured hippocampal neurons. As shown in Figure 2A,B, co-staining for NOX2 and NeuN revealed that hypoxia induced an upregulation of NOX2 staining, with a reduction in NeuN staining in the hippocampal CA1 region compared with the control group. Nevertheless, pre- and post-treatment with Dex effectively reduced the expression of NOX2 and increased neuronal survival following hypoxia (F_(3,12)_ = 16.68, *p* = 0.0001). Meanwhile, Dex treatment markedly suppressed the hypoxia-induced upregulation of NOX activity in the hippocampus (F_(3,12)_ = 38.65, *p* < 0.0001; Figure 2C). Moreover, Dex treatment reduced the quantities of the lipid peroxidation product MDA (F_(3,12)_ = 41.50, *p* < 0.001; Figure 2D) and the oxidative DNA damage marker 8-OHdG (F_(3,16)_ = 38.65, *p* < 0.0001; Figure 2E) while increasing the activity of SOD (F_(3,12)_ = 15.99, *p* = 0.0002; Figure 2F) and GSH-Px (F_(3,16)_ = 16.45, *p* < 0.0001; Figure 2G) in the hypoxic hippocampus.

**Figure 3:**
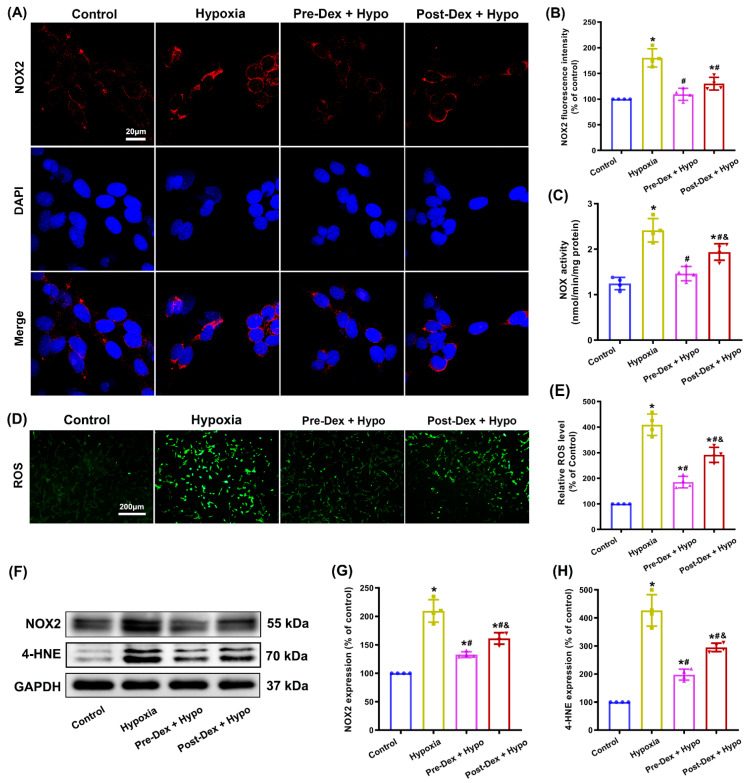
The effect of Dex on NOX2 activity and oxidative stress in cultured hippocampal neurons following hypoxia. (**A**) Representative confocal images stained for NOX2 (red) and DAPI (blue) in cultured hippocampal neurons. (**B**) The intensity of NOX2 fluorescence was quantified using ImageJ. (**C**) Cytochrome c reduction assays were used to measure NOX activity. (**D**) Intracellular ROS production was detected using the DCFH-DA probe. (**E**) The intensity of ROS fluorescence was analyzed using ImageJ. (**F**) NOX2 and 4-HNE protein levels were detected by Western blotting, and the relative expression levels of NOX2 (**G**) and 4-HNE (**H**) were analyzed using ImageJ. Results are expressed as mean ± SD (*n* = 4 in each group). Each data point in the bar graph is also presented. * *p* < 0.05 versus the Control group; ^#^ *p* < 0.05 versus the Hypoxia group; ^&^ *p* < 0.05 versus the Pre-Dex group.

**Figure 4:**
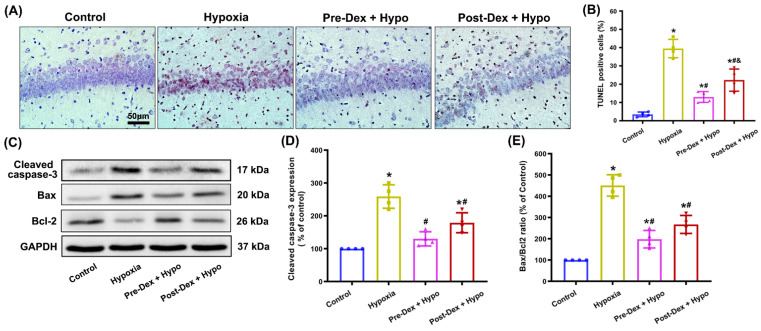
The effect of Dex on neuronal apoptosis in the hippocampus following neonatal hypoxia. (**A**) A TUNEL assay was used to evaluate neuronal apoptosis in the hippocampal CA1 region 1 d after neonatal hypoxia. (**B**) The apoptosis index (%) was calculated by dividing TUNEL-positive cells by the total number of all cells. (**C**) The levels of cleaved caspase-3, Bax, and Bcl-2 in the hippocampus were detected by Western blotting. (**D**,**E**) Quantitative analysis of the protein cleaved-caspase-3 (**D**) and Bax/Bcl-2 ratio (**E**). Results are expressed as mean ± SD (*n* = 4 in each group). Each animal data point in the bar graph is also presented. * *p* < 0.05 versus the Control group; ^#^ *p* < 0.05 versus the Hypoxia group; ^&^ *p* < 0.05 versus the Pre-Dex group.

In accordance with the in vivo results described above, our in vitro results indicated that exposure to hypoxia markedly increased the expression of the NOX2 protein in cultured hippocampal neurons; however, these increases in the expression of NOX2 were significantly attenuated by either pretreatment or post-treatment with Dex using immunofluorescence staining (F_(3,12)_ = 33.98, *p* < 0.0001; Figure 3A,B) and Western blotting assays (F_(3,12)_ = 66.85, *p* < 0.0001; Figure 3F,G). Moreover, NOX activity in the Dex-treated groups was much lower than that in the hypoxia group (F_(3,12)_ = 30.54, *p* < 0.0001; Figure 3C). As a biomarker of oxidative stress, cytotoxic 4-HNE contributes to the progression of neurodegeneration. We found that the levels of ROS and 4-HNE increased significantly in the hypoxia group, but in the Dex-treated groups, a significant reduction in ROS generation (F_(3,12)_ = 91.74, *p* < 0.0001; Figure 3D,E) and 4-HNE levels (F_(3,12)_ = 82.54, *p* < 0.0001; Figure 3F,H) was observed. These results indicate that the oxidative stress reduced by Dex might be attributable to the inhibition of NOX2 activation in hippocampal neurons following hypoxia.

### 3.2. Dex Reduced Mitochondrial Apoptosis in the Rat Hippocampus and Cultured Hippocampal Neurons Following Hypoxia

Since neuronal NOX2 activation-mediated oxidative stress usually contributes to apoptosis, next, we used a TUNEL assay and Western blotting to evaluate apoptosis in hippocampal neurons. The results indicated that the number of TUNEL-positive cells in the hippocampus was notably increased 1 d after neonatal hypoxia. Nevertheless, Dex treatment significantly reduced hypoxia-induced apoptosis, and Dex pretreatment appeared to have a greater effect than post-treatment (F_(3,12)_ = 52.10, *p* < 0.0001; Figure 4A,B). Additionally, the Western blotting results confirmed that neonatal hypoxia significantly increased the expression of the apoptosis-related protein cleaved caspase-3 and Bax, but reduced the expression of Bcl-2 compared with the control group. However, when compared with the hypoxia group, the levels of cleaved caspase-3 and Bax were decreased, and Bcl-2 was increased in the Dex-treated hypoxia groups (Figure 4C–E).

**Figure 5:**
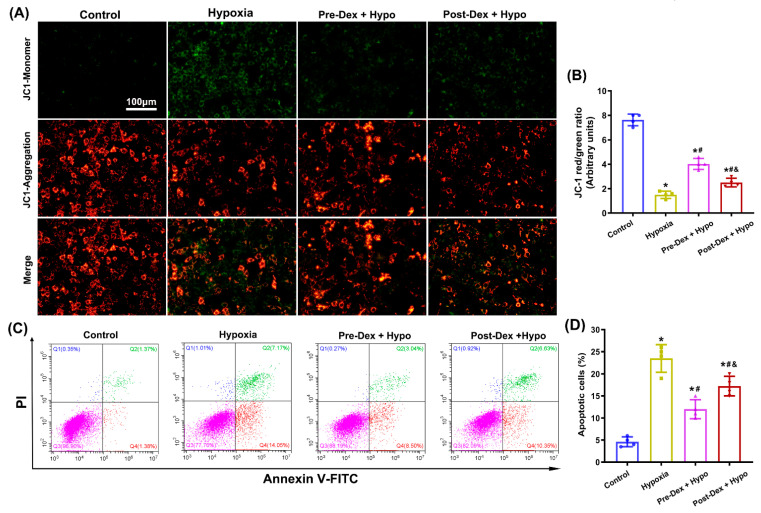
The effect of Dex on mitochondrial function and apoptosis in cultured hippocampal neurons following hypoxia. (**A**) Representative images of the MMP in hippocampal neurons using the mitochondrial probe JC-1. (**B**) The MMP is represented by the ratio of red to green JC-1 fluorescence. (**C**,**D**) Apoptosis was detected and analyzed using flow cytometry by staining cells with FITC-Annexin V/PI. Results are expressed as mean ± SD (*n* = 4 in each group). Each data point in the bar graph is also presented. * *p* < 0.05 versus the Control group; ^#^ *p* < 0.05 versus the Hypoxia group; ^&^ *p* < 0.05 versus the Pre-Dex group.

The MMP is a critical parameter for understanding mitochondrial function, and a decreased MMP indicates mitochondrial dysfunction and apoptosis [31]. Figure 5A,B show that the healthy control cells mainly presented red JC-1 aggregates, indicative of normal MMP. Increased green JC-1 monomers and reduced red JC-1 aggregates were observed in cells exposed to hypoxia, indicative of MMP loss. As expected, Dex pre- and post-treatment effectively caused a decrease in green JC-1 monomers and an increase in red JC-1 aggregates compared with those in hypoxic cells (F_(3,12)_ = 178.7, *p* < 0.0001), indicative of an improvement in the MMP. Moreover, flow cytometry analysis showed that hypoxia exposure induced obvious apoptosis in cultured hippocampal neurons, which was markedly suppressed by the Dex treatment ((F_(3,12)_ = 50.06, *p* < 0.0001; Figure 5C,D). These results suggested that Dex improved mitochondrial dysfunction and reduced apoptosis in hippocampal neurons following neonatal hypoxia.

### 3.3. Dex Alleviated Neuronal Injury and Cognitive Deficits in the Hippocampus Following Neonatal Hypoxia

**Figure 6:**
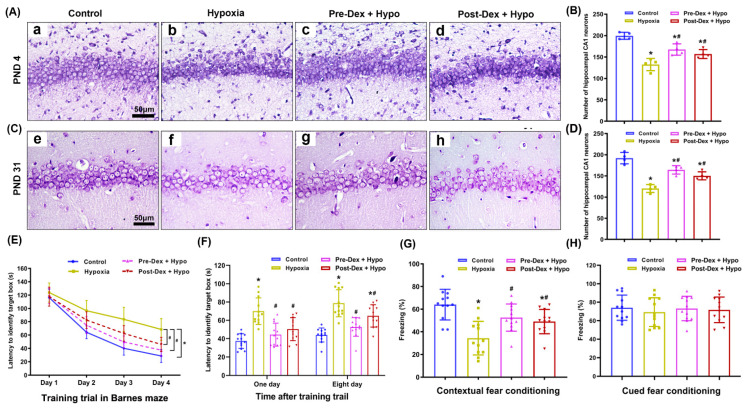
The effect of Dex on histopathological changes and cognitive function in the hippocampus following neonatal hypoxia. (**A**,**C**) Nissl staining was performed to evaluate hippocampal neuronal injury and morphological changes at 1 d (PND4) and 28 d (PND31) after neonatal hypoxia. (**B**,**D**) Quantification of the number of Nissl-positive cells present in the CA1 region of the hippocampus. (**E**) Performance with the Barnes maze test in training sessions. (**F**) Performance with the Barnes maze test in the memory phase. (**G**) Contextual fear conditioning test. (**H**) Cued fear conditioning test. Results are expressed as mean ± SD (*n* = 4 in each group for Nissl staining data; *n* = 12 in each group for Barnes maze and fear conditioning test data). Each animal data point in the bar graph is also presented. * *p* < 0.05 versus the Control group; ^#^ *p* < 0.05 versus the Hypoxia group.

In order to confirm the neuroprotection of Dex against hypoxic brain injury in neonates, Nissl staining and behavioral tests were further performed to investigate histopathological changes and neurological outcomes in the hippocampus after neonatal hypoxia. On PND4, Nissl staining revealed obvious neuropathological changes in the hippocampus in the hypoxia group compared with the control group, including Nissl body loss and nuclear shrinkage or disappearance. In contrast, Dex pretreatment and post-treatment both effectively attenuated hypoxia-induced neuronal injury and increased the number of Nissl bodies on PND4 (F_(3,12)_ = 22.23, *p* < 0.0001; Figure 6A,B). Moreover, we found that neonatal hypoxia induced long-term hippocampal neuronal injury lasting until PND31, as evidenced by a thin granular layer and a decreased density of pyramidal neurons compared with the control group. Dex treatment partially prevented morphological changes and increased the number of pyramidal neurons in the hippocampal CA1 region on PND31 (F_(3,12)_ = 31.17, *p* < 0.0001; Figure 6C,D).

During the Barnes maze training, the rats were able to identify target boxes faster with increased training sessions (Figure 6E). The results revealed that neonatal hypoxia was a significant factor affecting learning and memory. This effect could be attenuated by Dex treatment (F_(3,44)_ = 23.25, *p* < 0.0001). Notably, hypoxic rats took a much longer time than the control rats to reach the target box, which was also attenuated by the Dex treatment at 1 d (F_(3,44)_ = 16.05, *p* < 0.0001) and 8 d (F_(3,44)_ = 20.53, *p* < 0.0001) after the training sessions (Figure 6F). Moreover, rats in the hypoxia group showed less freezing behavior than the control mice during the contextual fear conditioning test, which was also attenuated by the Dex treatment (F_(3,44)_ = 10.78, *p* < 0.001; Figure 6G). Surprisingly, cued fear conditioning did not differ significantly among the four groups (F_(3,44)_ = 0.27, *p* = 0.85; Figure 6H). These results suggest that Dex effectively attenuated neuronal injury and hippocampus-dependent learning and memory deficits following neonatal hypoxia.

### 3.4. Dex Protected against Cognitive Deficits by Modulating NOX2-Derived Oxidative Stress in the Hippocampus Following Neonatal Hypoxia

**Figure 7:**
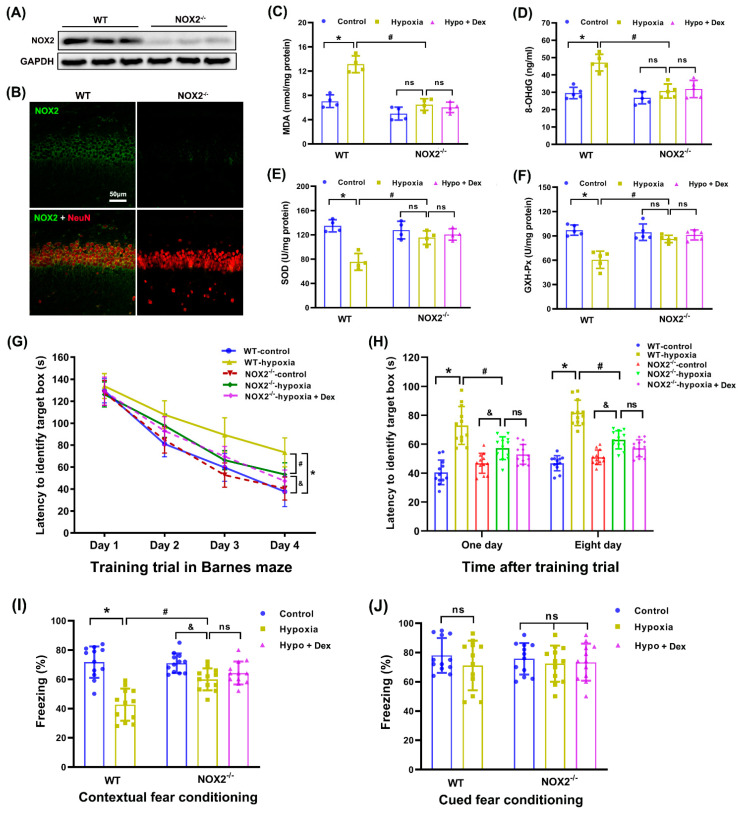
Modulation of Dex-induced neuroprotection in neonatal mice by NOX2 depletion. (**A**,**B**) Representative images of the genetic identification of NOX2^−/−^ neonatal mice via Western blotting and immunofluorescence assays in the hippocampus. (**C**) MDA levels were assessed using the TBA colorimetric method. (**D**) DNA 8-OHdG levels were assessed using an 8-OHdG ELISA kit. (**E**) SOD activity was assessed using the WST-1 method. (**F**) GSH-Px activity was assessed using the UV colorimetric assay. (**G**) Performance with the Barnes maze test in the training sessions. (**H**) Performance with the Barnes maze test in the memory phase. (**I**) Contextual fear conditioning test. (**J**) Cued fear conditioning test. Results are expressed as mean ± SD (*n* = 4 in each group for panel A and B; *n* = 4–5 in each group for panel C to F; *n* = 12 in each group for Barnes maze and fear conditioning test data). Each animal data point in the bar graph is also presented. * *p* < 0.05, ^#^ *p* < 0.05, ^&^ *p* < 0.05 for the comparison. ns, not significant.

In order to further determine whether NOX2 in the hippocampus is involved in Dex-induced neuroprotection against oxidative stress and the cognitive deficits caused by neonatal hypoxia, we used NOX2-deficient (NOX2^−/−^) mice to investigate its involvement in this process. Figure 7A,B show that NOX2 protein expression and immunoreactivity in the hippocampus were very low in NOX2^−/−^ neonatal mice compared with their WT littermates. We observed that NOX2 depletion significantly reduced the MDA (F_(4,15)_ = 35.42, *p* < 0.0001; Figure 7C) and DNA 8-OHdG (F_(4,20)_ = 18.12, *p* < 0.0001; Figure 7D) contents while increasing SOD (F_(4,15)_ = 15.01, *p* < 0.0001; Figure 7E) and GXH-Px (F_(4,20)_ = 17.16, *p* < 0.0001; Figure 7F) activities in the NOX2^−/−^ hippocampus compared with WT mice exposed to hypoxia. However, a combination of NOX2 depletion and Dex pretreatment did not achieve greater attenuation of oxidative stress than NOX2 depletion alone in hypoxic NOX2^−/−^ mice.

As described above, the mice were able to identify the target boxes faster with increased training sessions (Figure 7G). Compared with hypoxic WT mice, NOX2 depletion reduced the time to reach the target box at 1 d (F_(4,55)_ = 22.71, *p* < 0.0001) and 8 d (F _(4,55)_ = 54.82, *p* < 0.0001) after the training sessions (Figure 7H). Moreover, the freezing behavior in the contextual fear conditioning behavior test was significantly decreased in WT mice subjected to hypoxia, which was also attenuated by NOX2 depletion (F_(4,55)_ = 21.17, *p* < 0.0001; Figure 7I). Nonetheless, no experimental condition had an effect on the cued freezing behavior test (F_(4,55)_ = 0.53, *p* = 0.71; Figure 7J). Furthermore, a combination of NOX2 depletion and Dex pretreatment did not exert an additional beneficial effect on hypoxia-induced cognitive deficits compared with NOX2 depletion alone in hypoxic NOX2^−/−^ mice. These results indicate that the neuroprotective effect of Dex against oxidative stress and the long-term neurological deficits caused by neonatal hypoxia were dependent on NOX2 in the hippocampus.

### 3.5. Dex Suppressed ROS Production and Apoptosis by Modulating NOX2 in Cultured Hippocampal Neurons Following Hypoxia

**Figure 8:**
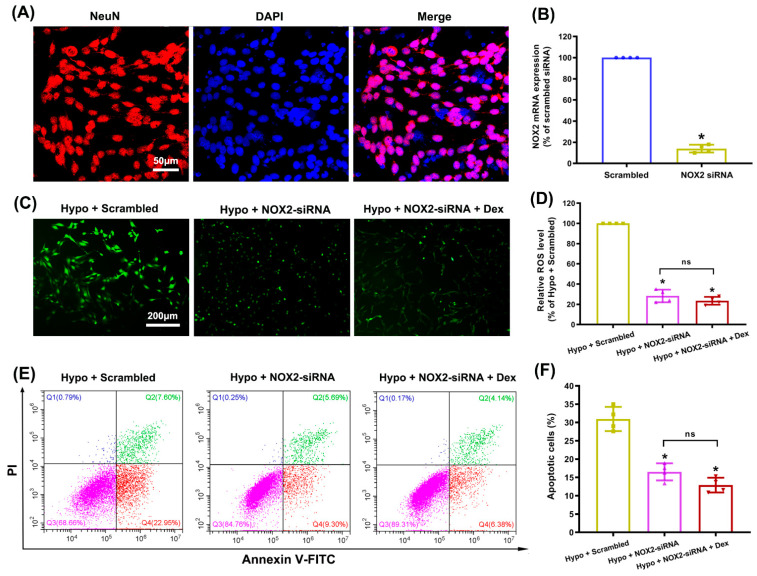
Modulation of Dex neuroprotection in cultured hippocampal neurons by NOX2 knockdown. (**A**) The purity of hippocampal neurons was evaluated by immunofluorescence detection with NeuN (red) and DAPI (blue). (**B**) The siRNA-mediated transfection efficiency of NOX2 was assessed using qRT-PCR. * *p* < 0.05 versus the Scrambled group. (**C**) Intracellular ROS production was detected using the DCFH-DA probe. (**D**) The intensity of ROS fluorescence was analyzed using ImageJ. (**E**,**F**) Apoptosis was detected and analyzed using flow cytometry by staining cells with FITC-Annexin V/PI. Results are expressed as mean ± SD (*n* = 4 in each group). Each data point in the bar graph is also presented. * *p* < 0.05 versus the Hypoxia + Scrambled group.

In order to more fully clarify the potential role of neuronal NOX2 in the Dex-mediated alleviation of ROS overproduction and apoptosis, we knocked down the expression of NOX2 in cultured hippocampal neurons using a NOX2-targeting siRNA. As shown in Figure 8A, the cultures had over 95% purity of hippocampal neurons, which met the experimental requirements. Next, qRT-PCR analysis indicated that the NOX2 mRNA levels in NOX2 siRNA-transfected cells significantly decreased compared with those in control cells transfected with scrambled siRNA (Figure 8B). We noticed that NOX2-siRNA significantly suppressed the hypoxia-induced overproduction of ROS in hippocampal neurons. However, the combination of NOX2-siRNA and Dex treatment did not achieve greater attenuation of ROS generation than NOX2-siRNA alone (*p* = 0.319; Figure 8C,D). Similarly, we found that a combination of NOX2-siRNA and Dex treatment did not achieve a greater reduction in apoptosis than NOX2-siRNA alone (*p* = 0.178; Figure 8E,F). The results fully demonstrated that the modulation of neuronal NOX2 activity was involved in the Dex-induced neuroprotection against hypoxic brain injury.

## 4. Discussion

It is well known that neonatal hypoxia can lead to serious neurological deficits in humans. Dex reportedly has an efficient antioxidant property, providing protective effects against hypoxic brain injury in neonates. This study explored whether the inhibition of overactivated neuronal NOX2 in the hippocampus was involved in the neuroprotection of Dex. Our results in hypoxia-injured cultured hippocampal neurons clearly show the potent role of Dex in suppressing NOX2 activation and ROS production, subsequently improving mitochondrial function and reducing apoptosis. Importantly, in vitro studies were corroborated in a rat model of neonatal hypoxic brain injury, in which the expression and activity of neuronal NOX2 obviously increased, accompanied by oxidative stress and apoptosis in the immature hippocampus, leading to hippocampal injury and cognitive deficits. Pretreatment or post-treatment with Dex effectively inhibited the activation of NOX2 and subsequent oxidative stress, alleviating mitochondrial apoptosis and neuronal injury, and ultimately improving neurological function. Notably, Dex did not exert significant neuroprotective effects against hypoxia-induced hippocampal injury in vitro or in vivo when NOX2 was knocked down. These results strongly indicated that Dex protects against oxidative damage and neurological deficits by modulating neuronal NOX2 activity in the hippocampus following neonatal hypoxia.

Perinatal asphyxia occurs in approximately 1–3 per 1000 live births at full term, and can reach 60% in premature neonates or those with a low birth weight [2]. In the present study, we established that experimental hypoxia in rodents on PND3 mimics perinatal hypoxia occurring in human preterm neonates born at 28–32 weeks, since the development of the rodent’s nervous system at 2–4 days of postnatal age is equivalent to the development of the human nervous system at 28–32 weeks of gestation [32]. The immature brain is extremely susceptible to damage by hypoxia due to its high oxygen requirements and poor antioxidative defense mechanisms [5]. Among all the brain tissues, the hippocampus is the area most sensitive to neonatal hypoxia–ischemia; therefore, the hippocampus was selected in this study [33,34]. We used the Barnes maze and contextual fear conditioning to test hippocampus-dependent tasks, and our results showed that Dex effectively attenuated long-term learning and memory dysfunction in neonatal rats following hypoxia. Notably, there were no obvious differences in the freezing rate among the groups in cued fear conditioning, indicating that the differences in cognitive function were not due to abnormalities in amygdala-dependent cued fear memory.

Apoptosis has been identified as a major pathological factor leading to delayed neuronal death and neuronal loss, which contribute to long-term neurological and cognitive deficits in the immature animal brain following hypoxic insult [19,35]. Thus, preventing or reducing neuronal apoptosis at an early stage is a promising strategy for neonatal hypoxic brain injury. In our in vivo studies, Dex was intraperitoneally administered either 30 min before or immediately after the pups were subjected to hypoxic insult. We then evaluated hippocampal neuronal apoptosis using the TUNEL assay and Western blotting of apoptosis-related proteins in neonatal rats following hypoxia and Dex treatment. Due to its high lipophilicity, Dex readily crosses the blood–brain barrier (BBB) and penetrates into brain tissue [36]. As expected, we found that Dex treatment effectively reduced neuronal apoptosis, decreased the Bax/Bcl-2 ratio, and cleaved caspase-3 expression in the hippocampus following neonatal hypoxia, and it appeared that pretreatment was more effective than post-treatment. The bcl-2 family of proteins has been reported to regulate apoptosis by either promoting or preventing it through a mitochondria-dependent pathway [35]. We further extended our study to an in vitro model, and we showed that Dex effectively improved hypoxia-induced mitochondrial dysfunction and apoptosis in cultured hippocampal neurons. The results revealed that Dex exerted potent neuroprotective effects against hippocampal neuronal apoptosis, likely through the mitochondrial pathway.

Mitochondrial ROS generation is thought to be an underlying cause of oxidative stress and apoptosis in the CNS following brain injury [37]. However, emerging experimental evidence has indicated that neuronal NOX2 is a main contributor to oxidative damage in various neurological diseases, including brain ischemia, brain trauma, and neurodegenerative disorders [11,12,13]. A novel finding in our present study was that Dex treatment effectively suppressed neuronal NOX2 activation and subsequent oxidative damage after hypoxia in vivo and in vitro. Considering that NOX2 and mitochondria exist in close proximity to each other in the neurons [38], there is a possibility that NOX2 produces “kindling” ROS that induce mitochondrial dysfunction, triggering an additional surge in free radicals from the mitochondria. These data strongly point to neuronal NOX2 as a potentially important therapeutic target for hypoxic brain injury in neonates.

NOX2, one of the NOX enzyme subunits, is expressed in the microglia and neurons, and contributes significantly to neuronal cell death and neurological deficits under pathological conditions [11]. Since the role of microglial NOX2 in neonatal hypoxic brain injury has been fully confirmed in our previous study [22], and neuronal NOX2 is reported to be expressed abundantly in the hippocampus, we focused our investigation on this target for the first time. Our results indicate that neonatal hypoxia increased the expression of NOX2, accompanied by the induction of marked neuronal loss and histopathological changes in the hippocampus, which were significantly reversed by the Dex treatment. In order to specifically examine the involvement of neuronal NOX2 in Dex-induced protection against hypoxic brain injury in neonates, we validated our findings by treating NOX2-deficient mice or NOX2-knockdown cultured hippocampal neurons with hypoxia and Dex. We observed that NOX2 deletion almost completely blocked hypoxia-induced upregulation of oxidative stress levels both in vivo and vitro, suggesting that NOX2 is a main source of ROS generation in hypoxia-induced oxidative stress in the immature hippocampus. Nevertheless, the protective effects of NOX2 deletion only partially reversed neuronal apoptosis and the hippocampus-dependent learning and memory deficits following hypoxia. These findings suggested that several mechanisms, including the NOX2/ROS pathway, had a role in the neurological deficits caused by neonatal hypoxia [3]. Moreover, a combination of NOX2 depletion and Dex treatment did not exert a greater beneficial effect on hypoxia-induced oxidative stress and cognitive deficits than NOX2 depletion alone, indicating that the neuroprotection of Dex against neurological deficits caused by neonatal hypoxia was NOX2-dependent in the hippocampus.

The main limitation of this study is that the detailed signaling pathways by which Dex regulates neuronal NOX2 activation were not fully determined. Previous studies by ourselves [15] and others [39,40] have already demonstrated that Dex potently inhibits an excessive influx of Ca^2+^ into the neurons through voltage-gated Ca^2+^ channels (VGCCs), NMDA receptors, or TRPM2 and TRPV1 channels during hypoxia and ischemia. Since NOX2 is a Ca^2+^-dependent enzyme that requires elevated Ca^2+^ levels for activation, we hypothesize that Dex-mediated neuronal NOX2 activation may be partly attributed to modulation of the intracellular Ca^2+^ concentration in the hippocampus following neonatal hypoxia. Additionally, in the present study, the dose of Dex (25 μg/kg, ip) used in vivo and the concentration of Dex (1 μM) used in vitro were determined on the basis of previous neuroprotection studies, including ours [22,25,26]. Nevertheless, more doses should be used to further investigate the dose–effect relationship in the future.

## 5. Conclusions

In conclusion, the present study investigated the effects of Dex on neuronal NOX2 activity and oxidative damage in neonatal animals subjected to hypoxic insult. Our study demonstrates for the first time that Dex effectively attenuates neuronal apoptosis and neurological deficits in animals neonatally subjected to hypoxia by modulating neuronal NOX2-derived oxidative stress in the immature hippocampus. Hence, the data point to neuronal NOX2 as a novel therapeutic target for hypoxic brain injury in neonates.

## Data Availability

Data are available from the corresponding author upon reasonable request.
